# Spheroid-cultured human umbilical cord-derived mesenchymal stem cells attenuate hepatic ischemia-reperfusion injury in rats

**DOI:** 10.1038/s41598-018-20975-0

**Published:** 2018-02-06

**Authors:** Yi Sun, Yang Wang, Liang Zhou, Yizhou Zou, Gengwen Huang, Ge Gao, Shi Ting, Xiong Lei, Xiang Ding

**Affiliations:** 1 Institute of Reproductive and Stem Cell Engineering, School of Basic Medical Science, Central South University, Changsha, 410078 China; 2 National Engineering and Research Center of Human Stem Cell, Central South University, Changsha, 410250 China; 3 Key Laboratory of Stem Cells and Reproductive Engineering, Ministry of Health, Changsha, 410250 China; 4 Department of Ophthalmology, Second Xiangya Hospital, Central South Univerisity, Changsha, 410011 China; 5 Department of Immunology, Xiangya School of Medicine, Central South University, Changsha, 410078 China; 6 Department of General Surgery, Xiangya Hospital, Central South University, Changsha, 410078 China; 7 Department of Laboratory Medicine, Xiangya School of Medicine, Central South University, Changsha, 410013 China; 8 Department of Organ Transplantation, Xiangya Hospital, Central South University, Changsha, 410078 China

## Abstract

Mesenchymal stem cell (MSC) transplantation is a promising treatment for ischemia-reperfusion injury (IRI). However, its effects on hepatic IRI were not consistent in the previous studies. 3D spheroid-cultured MSCs enhance their production of trophic and anti-inflammatory properties, but their effects on hepatic IRI remain unclear. In this study, we compared the 3D spheroid-cultured human umbilical derived MSCs (3D UC-MSCs) with 2D-cultured UC-MSCs (2D UC-MSCs) on treating hepatic IRI. The RNA sequencing data showed that suppression of cell mitosis, response to hypoxia, inflammation, and angiogenesis were the top genetic changes in 3D UC-MSCs compared with 2D UC-MSCs. Although both pro-inflammatory and anti-inflammatory genes were upregulated in the 3D UC-MSCs, the mRNA and protein of an RNase (ZC3H12A), which turnovers the mRNA of pro-inflammatory genes at the post-transcript level, were significantly upregulated in 3D UC-MSCs. 3D UC-MSCs reduced the secretion of many chemokines and growth factors, but increased the secretion of vascular endothelial growth factor. Compared with the vehicle and 2D UC-MSCs, 3D UC-MSCs significantly reduced hepatic IRI in rats, based on the plasma aminotransferase levels, liver damage scores, neutrophil infiltration, hepatocyte apoptosis and expression of inflammation-associated genes. These findings suggest that 3D UC-MSCs therapy is a promising treatment for hepatic IRI.

## Introduction

The hepatic ischemia-reperfusion injury (IRI) is a leading cause of primary graft dysfunction after liver transplantation and is associated with poor 1-year graft and patient survival rates of only 55% and 68%, respectively, compared with 90% and 93% for the remainder^1^. Although some strategies, such as ischemic preconditioning and application of pharmacological agents, seemed to be promising in laboratory experiments, only few of them have been tested in clinical randomized controlled trials^2–4^, and the results were not satisfactory enough to be acceptable in clinical routine.

Current advances in regenerative medicine showed that mesenchymal stem cell (MSC) transplantation seemed to be a promising treatment for IRI^5^. MSCs represent a heterogeneous population of adult fibroblast-like multipotent cells which can replicate and differentiate to multiple cell lineage pathways. They are well suitable for cell therapy as they express few HLA class I and no HLA class II molecules^6–8^, which enable them to evade allogeneic immune response after transplantation. MSC therapy has shown beneficial effects on IRI of heart, intestine, kidney, and brain^5,9–12^. Although the exact mechanism is not fully understood, it seems that paracrine of trophic and anti-inflammatory cytokines, including basic fibroblast growth factor (bFGF), vascular endothelial growth factor (VEGF), hepatocyte growth factor (HGF), and interleukin(IL)-10, plays an important role in MSC therapy^10,13–17^. The effect of MSC therapy for hepatic IRI had been studied by several groups. However, the results were not consistent. While some studies showed that MSC therapy could prevent hepatic IRI by suppressing inflammatory responses, oxidative stress and apoptosis^18–21^, others failed to reduce hepatic IRI with the same kind of MSCs.22–24 One reason for the failure might be that MSCs were short lived and did not migrate beyond the lungs after intravenous infusion^22–24^. Another reason might be that MSCs could be either pro-inflammatory or anti-inflammatory depending on the levels of inflammatory cytokines^25^, and which receptor was activated^26^.

Recently, several groups reported that aggregation of MSCs into 3-dimensional (3D) spheroids could greatly enhance their production of trophic and anti-inflammatory properties, such as tumor necrosis factor-alpha stimulated gene/protein 6 (TSG-6), prostaglandin E2, VEGF, and bFGF^16,27–29^. Moreover, the 3D culture of MSCs resulted in 75% reduction of individual cell volume, which significantly improved their ability of trafficking through the lung microvasculature^28^. 2D cultured MSCs lost their expression of some key receptors, such as C-X-C chemokine receptor type 4, for cell migration. While 3D culture could restore the expression of these receptors, which were critical for MSCs homing to the injury site^30,31^. The 3D MSCs have been reported to be beneficial for liver fibrosis and hepatitis^32,33^, but their effect on hepatic IRI remains largely unknown. Different kind of MSCs exhibits different immunobiological properties, among which umbilical cord lining MSCs (UC-MSCs) have especially low immunogenicity compared with other extraembryonic tissue–derived MSCs^34^. UC-MSCs showed the slowest rejection kinetics and lowest activation rate of T cells in an *in vivo* transplantation experiment^35^, but their effect on hepatic IRI has not been fully tested. In this study, we aimed to study the benefit of 3D UC-MSCs for treating hepatic IRI compared with 2D UC-MSCs, and the potential mechanisms.

## Results

### **A**ggregation of human UC-MSCs into spheroids caused significant changes in RNA transcription

**Figure 1:**
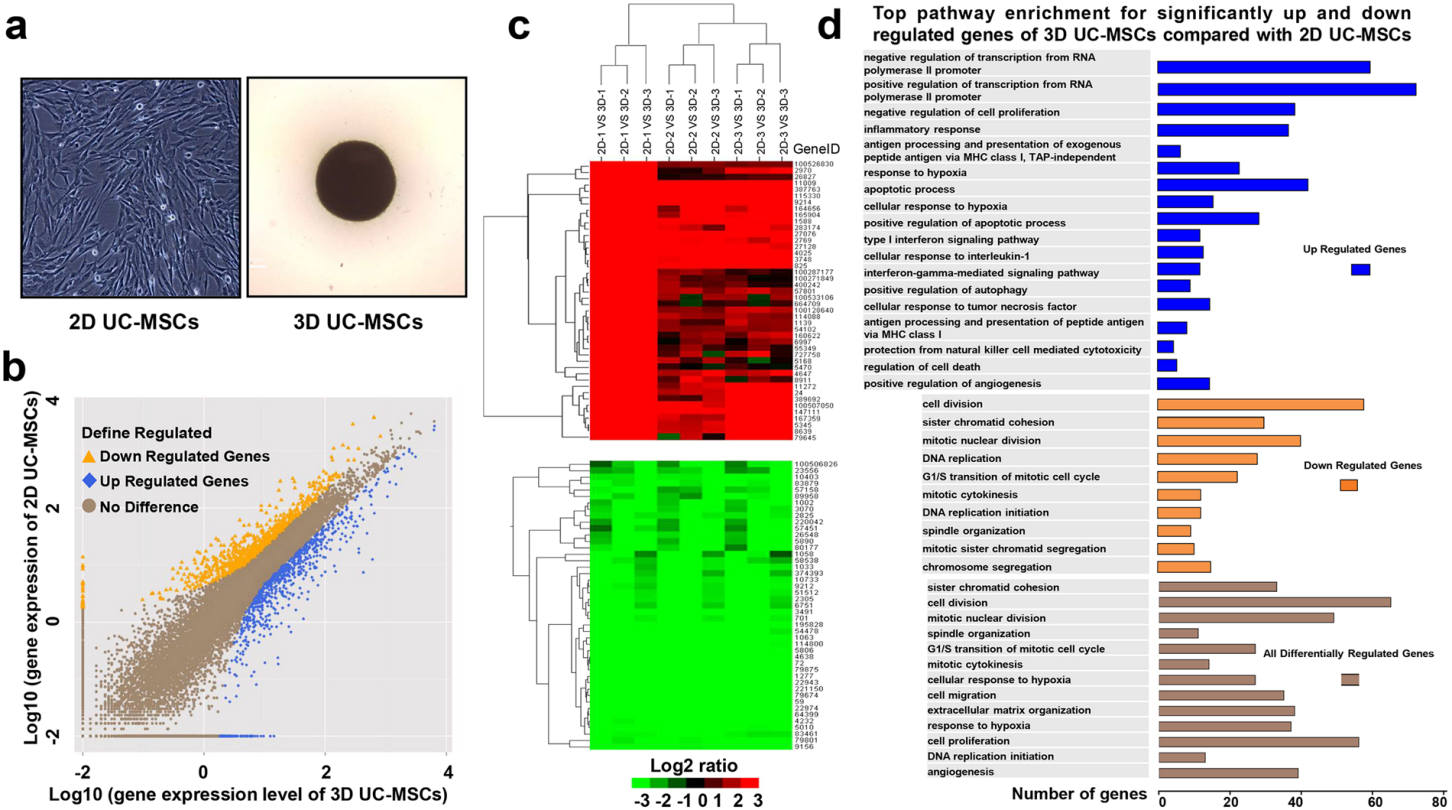
The 3D-spheroid culture of human UC-MSCs caused significant changes in gene expression. (**a**) Representative images of 2D- and 3D-cultured UC-MSCs under the phase-contrast microscope. (**b**) The difference of gene expression between 2D and 3DUC-MSCs was analyzed by RNA-sequencing; altogether 831 genes (blue) were significantly upregulated and 788 genes (yellow) were significantly downregulated in 3D UC-MSCs compared with 2D UC-MSCs (probability >80% and |log_2_(fold of gene expression change)|≥1). (**c**) The representative global view of gene expression changes between 2D UC-MSCs (n = 3, samples named 2D-1, 2D-2, 2D-3) and 3D UC-MSCs (n = 3, samples named 3D-1, 3D-2 and 3D-3) by hierarchical clustering analysis. The clusters in black denote no significant difference between 2D and 3D UC-MSCs, while the clusters in red or green denote transcripts which are more or less abundant compared to 2D UC-MSCs. The intensity of the colors is proportional to a log2 ratio of the fold of gene changes. (**d**) The gene ontology analysis showed the list of bioprocess terms most strongly enriched with the differentially regulated genes between 3D and 2D UC-MSCs. Each bar is proportional to the number of genes enriched in the bioprocess. Abbreviations: UC-MSCs, umbilical cord-derived mesenchymal stem cells.

During the process of cell culture, the time-lapse microscopy demonstrated that UC-MSCs cultured in hanging drops formed a loose network at first, and then, gradually coalesced into a single central spheroid along the lower surface of the drop (Fig. 1a), The RNA sequencing results showed that among the 19219 screened genes, altogether 831 genes were significantly upregulated and 788 genes were significantly downregulated in 3D UC-MSCs compared with 2D UC-MSCs (probability >80% and |log_2_(fold of gene expression change)|≥1) (Fig. 1b). The function clustering and gene ontology analysis (Fig. 1c,d) demonstrated that the significantly up-regulated genes were aggregated in various biological processes, including negative regulation of cell proliferation, inflammatory response, response to hypoxia, positive regulation of apoptotic process, positive regulation of autophagy, cellular response to tumor necrosis factor, and positive regulation of angiogenesis. While, the down-regulated genes in 3D UC-MSCs were mainly topped in the mitotic biological processes, including cell division, sister chromatid cohesion, mitotic nuclear division, DNA replication, G1/S transition of the cell cycle, mitotic cytokinesis, replication initiation, and spindle organization, indicating that 3D culture significantly inhibited cell proliferation. Moreover, the gene ontology analysis using all the differentially regulated genes also revealed that biological processes in cell mitosis, response to hypoxia and angiogenesis were still the top changes in 3D UC-MSCs compared with 2D UC-MSCs (Fig. 1d).

We further analyzed the genes enriched in the positive regulation of angiogenesis process and found that altogether 15 genes were significantly upregulated in 3D UC-MSCs (Supplementary Table 1). These genes included angiopoietin like 4, chitinase 3 like 1, adrenomedullin, and heme oxygenase 1, which were topped in the most differentially regulated genes (probability ≥95%, and |log_2_(fold of gene expression change)|>3.7) of all. Among the genes enriched in the inflammatory response (Supplementary Table 2), many pro-inflammatory genes were significantly upregulated in 3D UC-MSCs compared with 2D UC-MSCs, these included IL-1A, IL-1B, IL-6, IL-11, IL-24, IL-32, IL-33, chemokine (C-C motif) ligand (CCL) 2, CCL7, CCL20, chemokine (C-X-C motif) ligand (CXCL)3, CXCL5, and CXCL8. However, some anti-inflammatory genes were significantly upregulated in 3D UC-MSCs as well. These genes include the TSG-6, TNF-α-induced protein 8, and prostaglandin-endoperoxide synthase. Furthermore, the zinc finger CCCH-type containing 12A (ZC3H12A), an important anti-inflammatory gene whose product could inhibit inflammation by destabilizing inflammation-related mRNAs, such as IL-1, IL-2, IL-6, CXCL1, CXCL2, and CXCL3, was significantly upregulated in 3D UC-MSCs.

### 3D UC-MSCs increased the expression of ZC3H12A protein and VEGF secretion, but reduced the production of pro-inflammatory chemokines

**Figure 2:**
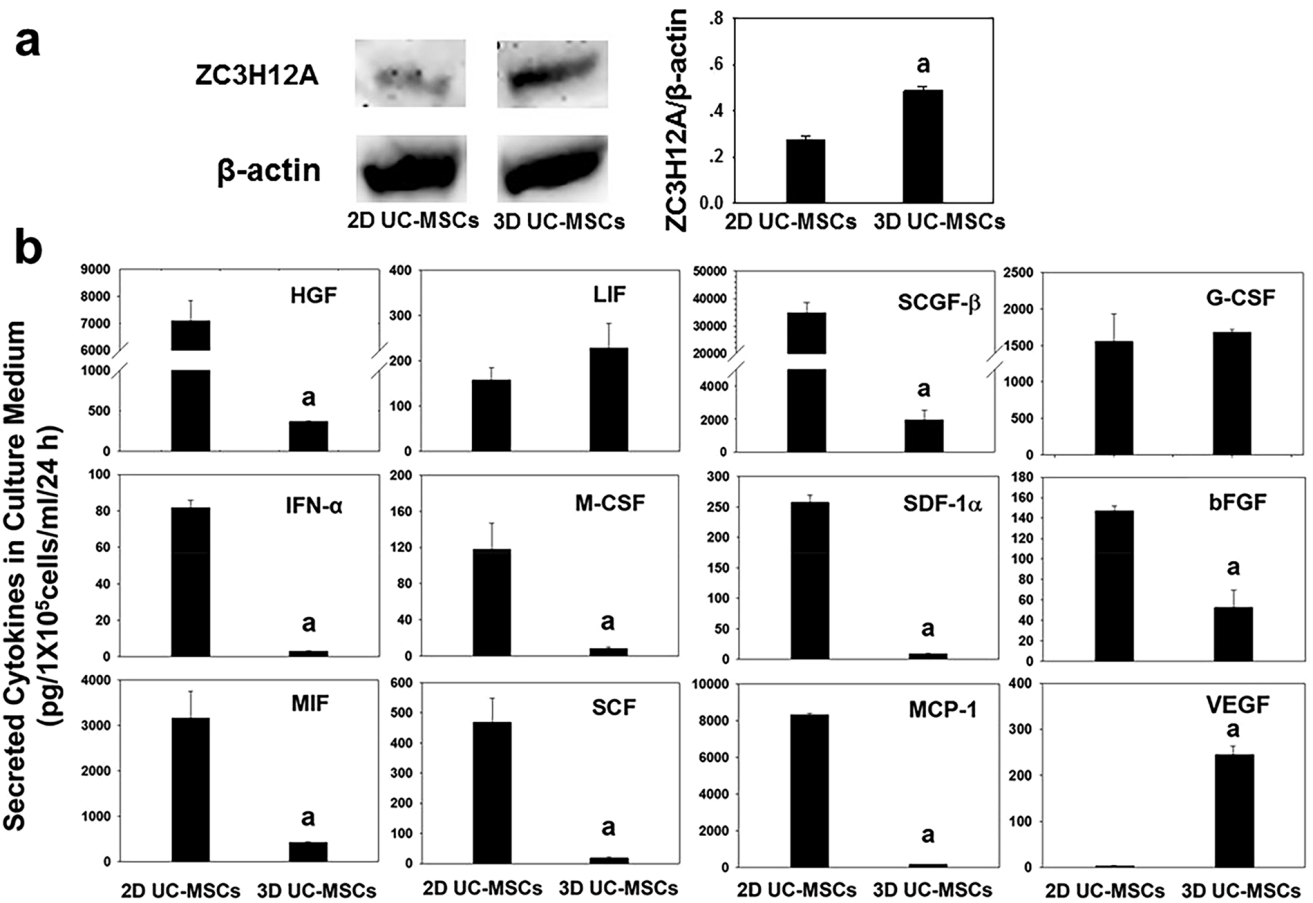
The 3D spheroid-culture of human UC-MSCs significant upregulated ZC3H12A protein expression and induced profound changes in cytokine production compared with 2D UC-MSCs. (**a**) The expression of ZC3H12A and actin proteins in 2D- and 3D-cultured UC-MSCs was detected by Western blotting. The representative blots from same gel were cropped and showed in the picture. The Western Blotting experiments were performed twice to verify the result consistency. The full-length gels and blots are included in Supplementary Figure 2. The expression of ZC3H12A protein was significantly increased in 3D UC-MSCs compared with 2D UC-MSCs. Data are presented as means ± SE (n = 4); ^a^*P* < 0.05 compared with 2D UC-MSCs. (**b**) The UC-MSCs cultures are as described in materials and methods. The culture medium was collected at 72 h time points, the concentrations of cytokines, as determined by multiplex-microbead immunoassay, are shown. Data are presented as means ± SE (n = 3); ^a^*P* < 0.05 compared with 2D UC-MSCs. Abbreviations: ZC3H12A, the zinc finger CCCH-type containing 12A; HGF, hepatocyte growth factor; LIF, leukemia inhibitory factor; SCGF-β, stem cell growth factor-β; G-CSF, granulocyte-colony stimulating factor; IFN-α, interferon-α; M-CSF, macrophage-colony stimulating factor; SDF-1α, stromal cell-derived factor 1α; bFGF, basic fibroblast growth factor; MIF, macrophage migration inhibitory factor; SCF, stem cell factor; MCP-1, Monocyte chemoattractant protein-1; VEGF, vascular endothelial growth factor; UC-MSCs, umbilical cord-derived mesenchymal stem cells.

To further investigate whether the significant changes in RNA expression of 3D UC-MSCs resulted in a corresponding alteration of ZC3H12A protein and cytokine production, we utilized the Western Blotting and Multiplex-Microbead Immunoassay to analyze the ZC3H12A protein and the cytokines produced by 2D and 3D UC-MSCs. As shown in Fig. 2a, the ZC3H12A protein level was significantly higher (0.47 ± 0.018 VS 0.26 ± 0.019, *P* < 0.05) in 3D UC-MSCs compared with 2D UC-MSCs. The results of Multiplex-Microbead Immunoassay (Fig. 2b) showed that 2D UC-MSCs secreted a lot of cytokines including macrophage migration inhibitory factor (MIF), monocyte chemoattractant protein-1 (MCP-1), interferon-α (IFN-α), leukemia inhibitory factor (LIF), stromal cell-derived factor 1α (SDF-1α), stem cell growth factor-β (SCGF-β), macrophage-colony stimulating factor (M-CSF), stem cell factor (SCF), and granulocyte-colony stimulating factor (G-CSF). Among these cytokines, the levels of SCGF-β (34834.1 ± 3738.2 pg/ml/1 × 10^5^ MSCs/24 h), MCP-1 (8307.2 ± 107.0 pg/ml/1 × 10^5^ MSCs/24 h), and MIF (3157.2 ± 591.3 pg/ml/1 × 10^5^ MSCs/24 h) were the highest in the conditioned medium. The 3D UC-MSCs significantly reduced their secretion of cytokines such as IFN-α, M-CSF, and SDF-1α. The concentrations of SCGF-β (1952.5 ± 578.2 pg/ml/1 × 10^5^ MSCs/24 h), MCP-1 (169.0 ± 3.8 pg/ml/1 × 10^5^ MSCs/24 h), and MIF (425.3 ± 11.3 pg/ml/1 × 10^5^ MSCs/24 h) were also decreased dramatically in 3D UC-MSCs conditioned medium. The secretion of LIF (157.6 ± 26.8 VS 228.3 ± 54.4 pg/ml/1 × 10^5^ MSCs/24 h, respectively) and G-CSF (1554.4 ± 380.5 VS 1684.8 ± 31.9 pg/ml/1 × 10^5^ MSCs/24 h, respectively) was remained similar between 2D and 3D UC-MSCs. In the aspect of trophic factors, both 2D and 3D UC-MSCs barely produced any platelet-derived growth factor-BB. The 2D UC-MSCs produced a high level of HGF (7103.6 ± 732.5 pg/ml/1 × 10^5^ MSCs/24 h) and some bFGF (147.0 ± 5.1, pg/ml/1 × 10^5^ MSCs/24 h), but the VEGF was almost undetectable. The 3D UC-MSCs secreted a large amount of VEGF (245.56 ± 17.87 pg/ml/1 × 10^5^ MSCs/24 h), but the secretion of HGF (367.7 ± 8.9 pg/ml/1 × 10^5^ MSCs/24 h) and bFGF (52.77 ± 16.93 pg/ml/1 × 10^5^ MSCs/24 h) was significantly reduced compared with 2D UC-MSCs.

### 3D UC-MSCS transplantation had a better therapeutic effect than 2D uc-mscs in treating hepatic iri in rats

**Figure 3:**
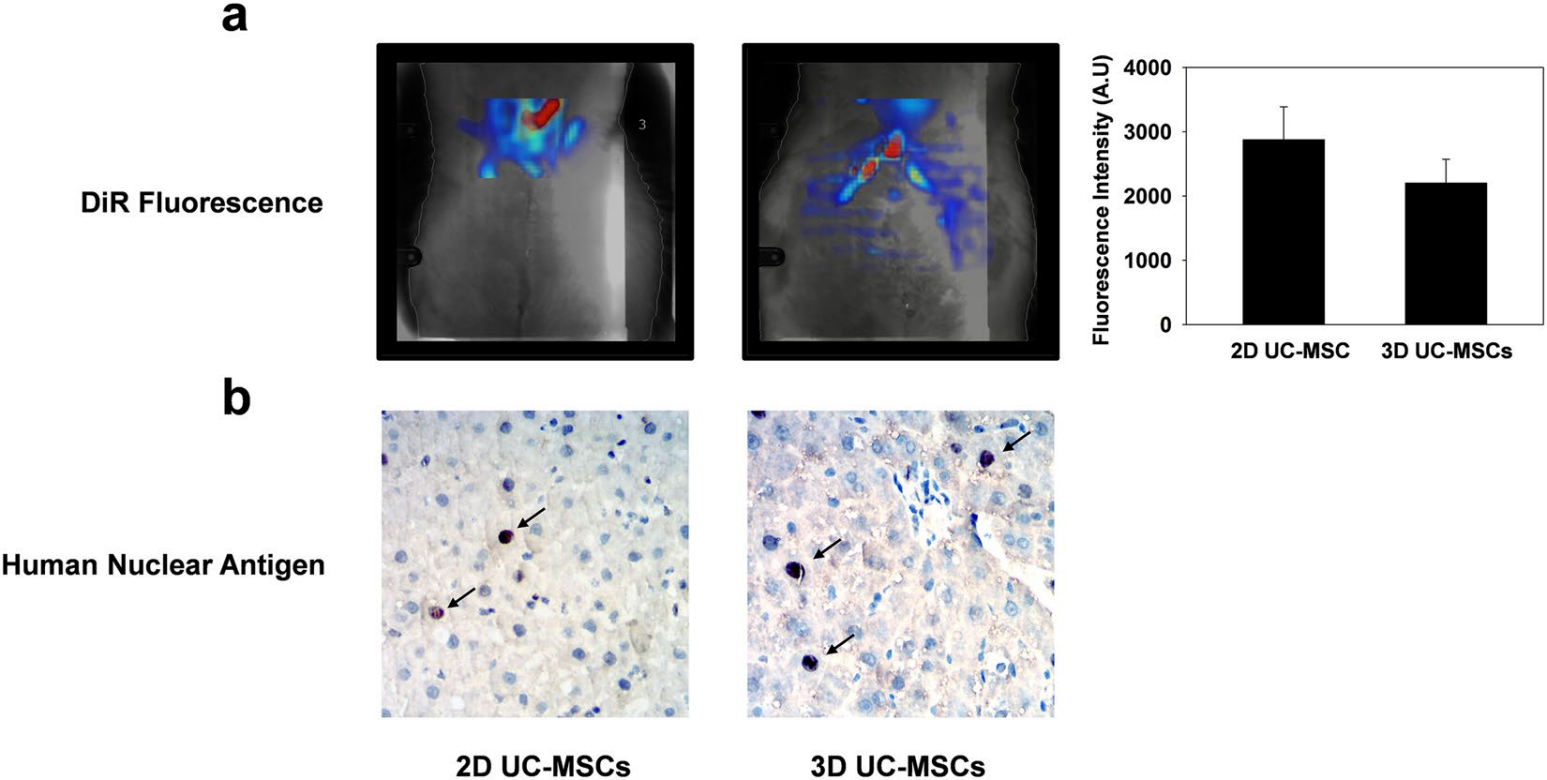
2D and 3D cultured UC-MSCs could migrate into the ischemic damaged liver after intraperitoneal injection. (**a**) The *in vivo* imaging showed that both 2D and 3D UC-MSCs could aggregate into the ischemia-damaged liver at 6 h after intraperitoneal injection by observing the fluorescent signal (red) from DiR labeled UC-MSCs. The fluorescence intensity of were not statistically different between 2D and 3D UC-MSCs. Data are presented as means ± SE (n = 3). (**b**) The migration of UC-MSCs into the liver was further confirmed by immunohistochemical staining (x400) of human nuclear antigen (arrows). Abbreviations: DiR, 1,1′-dioctadecyltetramethyl indotricarbocyanine Iodide; UC-MSCs, umbilical cord-derived mesenchymal stem cells.

Altogether 3 × 10^6^ 2D UC-MSCs or 3D UC-MSCs were administrated through intraperitoneal injection at the end of surgery. The *in vivo* imaging showed that the fluorescent signal from 2D and 3D UC-MSCs could aggregate into the liver at 6 h after administration (Fig. 3a). However, there was no statistical difference in hepatic fluorescence intensity between 2D and 3D UC-MSCs treatment groups (2875.25 ± 514.51 A.U. VS 2204.85 ± 368.15 A.U, *P* > 0.05). To confirm that the live UC-MSCs migrated into the liver, the staining of human nuclear antigen was performed in rat liver tissues. The results showed that the human nuclear antigen in 2D and 3D UC-MSCs could be detected in the liver at 6 hours after hepatic IRI (Fig. 3b).

**Figure 4:**
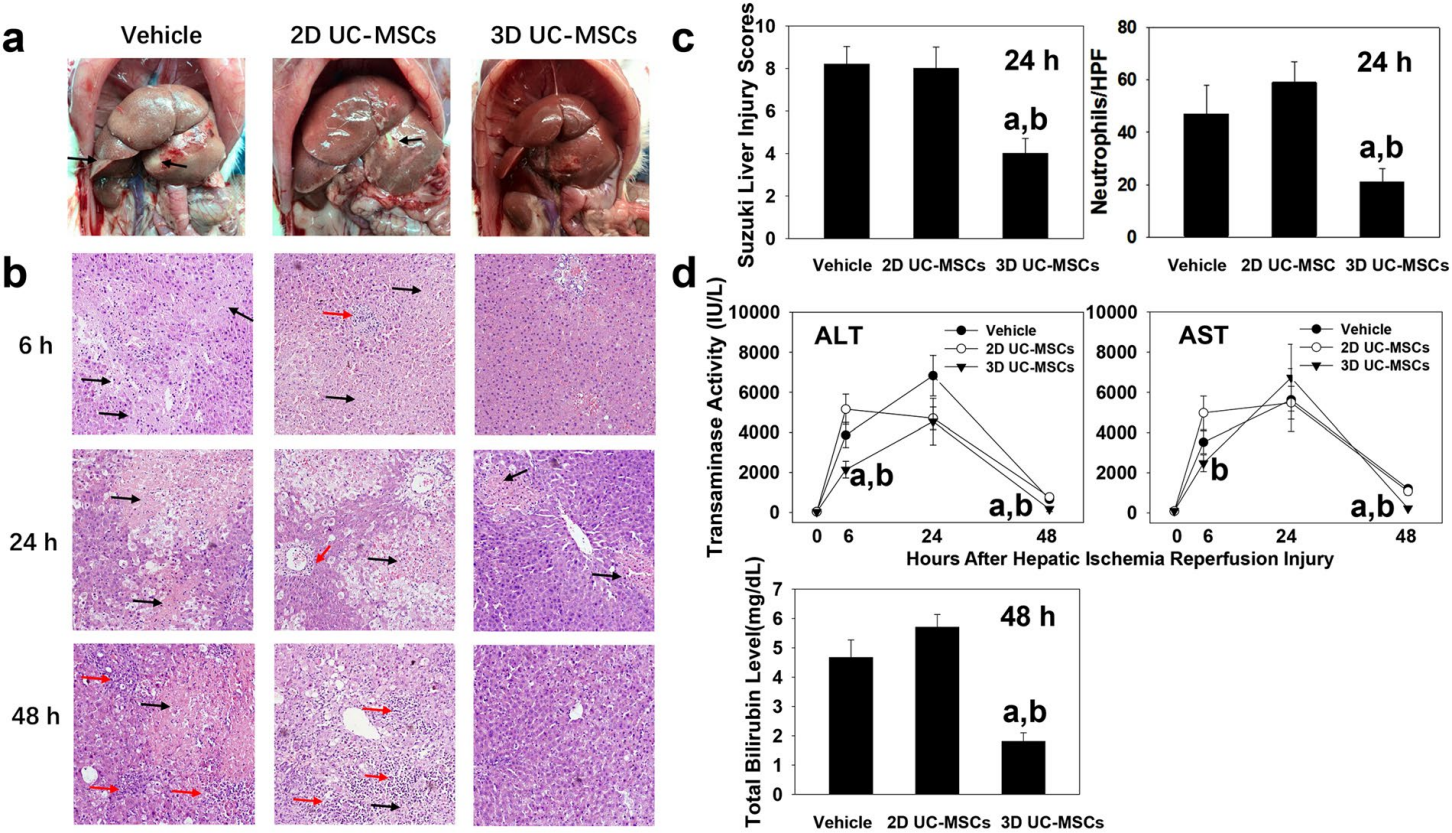
3D UC-MSCs attenuated the hepatic IRI in rats. (**a**) The representative gross looking of rat livers at 48 h after 70% hepatic IRI. The vehicle (left) and 2D UC-MSCs (middle) treatment groups had significant edema and necrosis (black arrow), while 3D UC-MSCs (right) treatment group only had mild edema. (**b**) The representative photomicrographs (×100) depicting liver damages at 6 h, 24 h, and 48 h after hepatic ischemia in the vehicle (left), 2D UC-MSCs (middle), and 3D UC-MSCs (right) treatment groups. Significant hepatic inflammation (red arrow) and necrosis (black arrow) could be seen in the vehicle and 2D UC-MSCs treatment group, while 3D UC-MSCs treatment group had much less tissue necrosis and inflammation. (**c**) liver injury was evaluated by injury scores and hepatic neutrophil infiltration. The 3D UC-MSCs treatment group had much lower injury score and less neutrophil infiltration compared with vehicle and 2D UC-MSCs treatment group. (**d**) Quantitation of the levels of plasma transaminases and plasma total bilirubin at various time points after hepatic IRI. Data are presented as means ± SE (n = 4–6). ^a^*P* < 0.05 compared with vehicle treatment group; ^b^*P* < 0.05 compared with 2D UC-MSCs treatment group. Abbreviations: ALT, alanine aminotransferase; AST, aspartate aminotransferase; UC-MSCs, HPF, high power fields (400×); umbilical cord-derived mesenchymal stem cells.

The pathological changes of hepatic IRI were investigated in this study. The results showed that 2D UC-MSCs treatment did not attenuate the hepatic IRI compared with the vehicle group, while 3D UC-MSCs treatment significantly reduced the hepatic necrosis and inflammation both in gross observation (Fig. 4a) and in microscopic sections (Fig. 4b). The Suzuki liver injury score (Fig. 4c left panel) in 3D UC-MSCs treatment group at 24 hours after hepatic IRI was 4.00 ± 0.71, which was significantly lower than the vehicle (8.20 ± 0.84, *P* < 0.05) and 2D UC-MSCs treatment groups (8.00 ± 1.01, *P* < 0.05). The chloroacetate esterase staining (Supplementary Figure 1) also revealed that much less neutrophil infiltration in the 3D UC MUSCs treatment group compared with the vehicle and the 2D UC-MSCs treatment groups (Fig. 4c right panel).

The liver injury was assessed by measuring alanine aminotransferase (ALT), aspartate aminotransferase (AST), and total bilirubin in the rat plasma. The plasma AST and ALT were within normal ranges in the absence of hepatic IRI. Hepatic ischemia for 90 minutes significantly increased AST and ALT levels after surgery. 2D UC-MSCs administration did not significantly alter the AST and ALT levels compared with the vehicle group at all time points; however, the 3D UC-MSCs administration significantly reduced the ALT levels at 6 h (2140.45 ± 416.00 IU/L) and 48 h time points (168.47 ± 62.69 IU/L) compared with the vehicle (3871.08 ± 628.53 IU/L at 6 h and 658.27 ± 79.86IU/L at 48 h) and 2D UC-MSCs (5157.88 ± 749.70 at 6 h and 772.30 ± 49.50 IU/L at 48 h) treatment groups (*P* < 0.05). The AST levels in the 3D UC-MSCs treatment group were significantly lower than the 2D UC-MSCs treatment group.at 6 h (2477.58 ± 419.48 VS 4980.30 ± 833.86 IU/L, respectively; *P* < 0.05) and 48 h (224.33 ± 17.21 VS 1077.70 ± 86.82 IU/L, respectively; *P* < 0.05). Although plasma ALT and AST levels in all groups were significantly decreased at 48 h after hepatic IRI compared with the levels at 6 h and 24 h time points, the total bilirubin levels were significantly higher in the vehicle (4.68 ± 0.60 mg/dL) and 2D UC-MSCs (5.70 ± 0.44 mg/dL) treatment groups compared with the 3D UC-MSCs treatment group (1.80 ± 0.30 mg/dL, *P* < 0.05) at 48 h (Fig. 4d).

### 3D UC-MSCS treatment did not promote hepatic regeneration but significantly inhibited apoptosis

**Figure 5:**
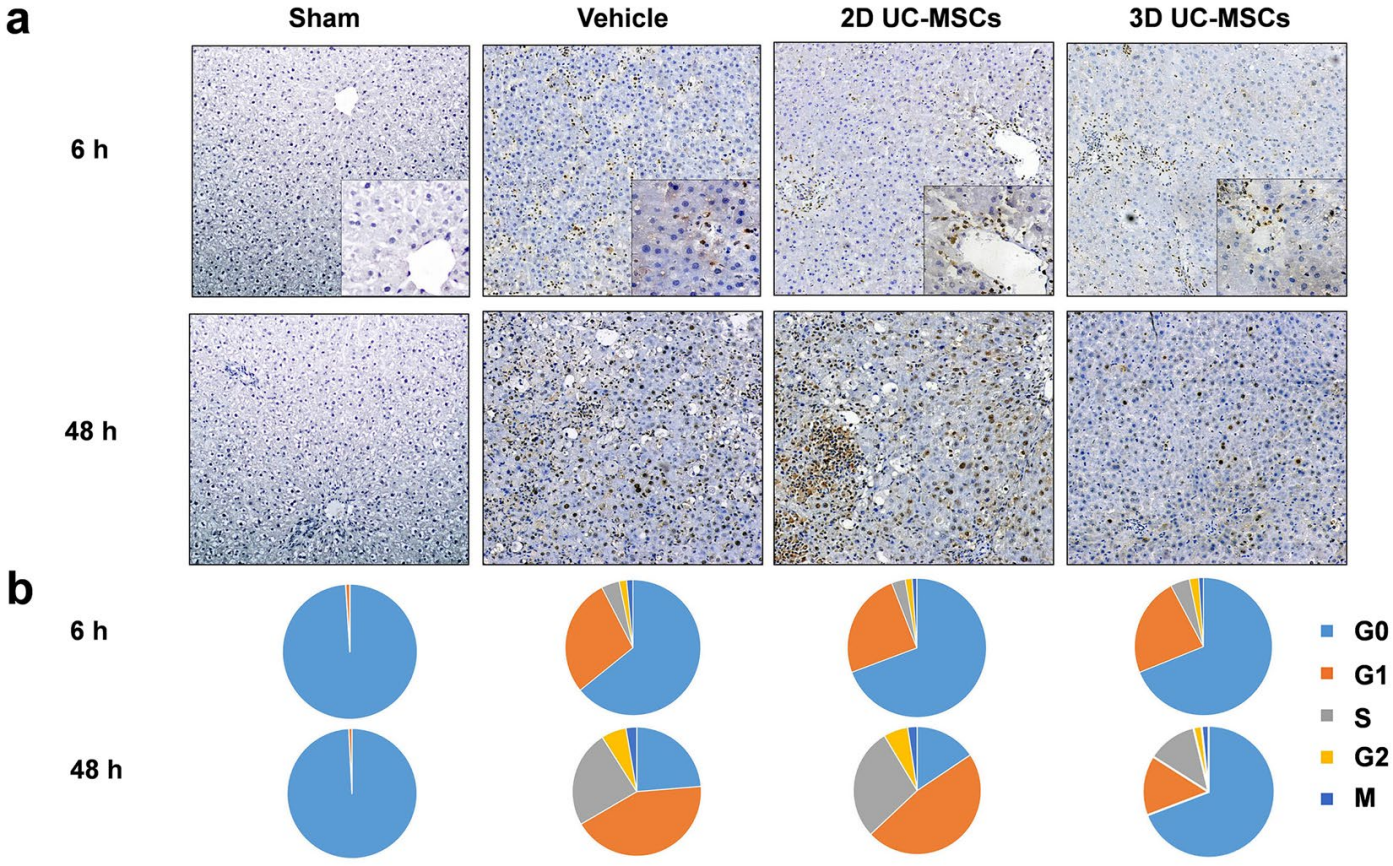
Evaluation of liver regeneration in non-necrotic areas by PCNA immunostaining. (**a**) Representative photomicrographs (×100) of PCNA staining at 6 h and 48 h after hepatic IRI in Sham surgery (left), vehicle treatment (left middle), 2D UC-MSCs treatment (right middle) and 3D UC-MSCs treatment (right) groups. Few hepatocytes were PCNA positive in the Sham surgery group at 6 and 48 h time points. The hepatic IRI induced a significant proliferation of hepatocytes in the non-necrosis areas of liver. Some inflammatory cells were also PCNA positive as showed in higher magnification (×400). (**b**) Cell cycle progression (per 1,000 hepatocytes) at different time points according to the PCNA staining pattern. At 48 h after hepatic IRI, the vehicle and 2D UC-MSCs treatment group had significantly more hepatocytes entering cell cycle compared with 3D UC-MSCs treatment group, which had much more hepatocytes staying in the G_0_ phase. Abbreviations: PCNA, proliferating cell nuclear antigen; IRI, ischemia-reperfusion injury; UC-MSCs, umbilical cord-derived mesenchymal stem cells.

The liver regeneration after hepatic IRI was analyzed by immunostaining of proliferating cell nuclear antigen (PCNA) (Fig. 5a). In the sham surgery group, few hepatocytes entered cell cycle at all time points. In the vehicle group with IRI, the number of PCNA positive cells increased significantly in the non-necrotic areas, most of which were in the G_1_ phase at 6 h after hepatic IRI, and then, more cells entered S, G_2_ and M phases at 24 h and 48 h time points. 2D and 3D UC-MSCs treatment did not significantly alter the rate of entry into the cell cycle at 6 h and 24 h compared with the vehicle group. Indeed, 3D UC-MSCs treatment significantly reduced the number of hepatocytes remained in proliferation at 48 h after hepatic IRI (Fig. 5b). For example, there were much more hepatocytes returned to the G_0_ phase (695 ± 95 per 1,000 hepatocytes) in the 3D UC-MSCs group compared with the vehicle (236 ± 54 per 1,000 hepatocytes, *P* < 0.05) and 2D UC-MSCs groups (161 ± 35 per 1,000 hepatocytes, *P* < 0.05). While there were much fewer hepatocytes entered G_1_ (144 ± 31 per 1,000 hepatocytes) and S phases (113 ± 28 per 1,000 hepatocytes) in the 3D UC-MSCs group compared with the vehicle (422 ± 51 and 238 ± 44 per 1,000 hepatocytes, respectively, *P* < 0.05) and 2D UC-MSCs (465 ± 63 and 279 ± 33 per 1,000 hepatocytes, respectively, *P* < 0.05) groups.

**Figure 6:**
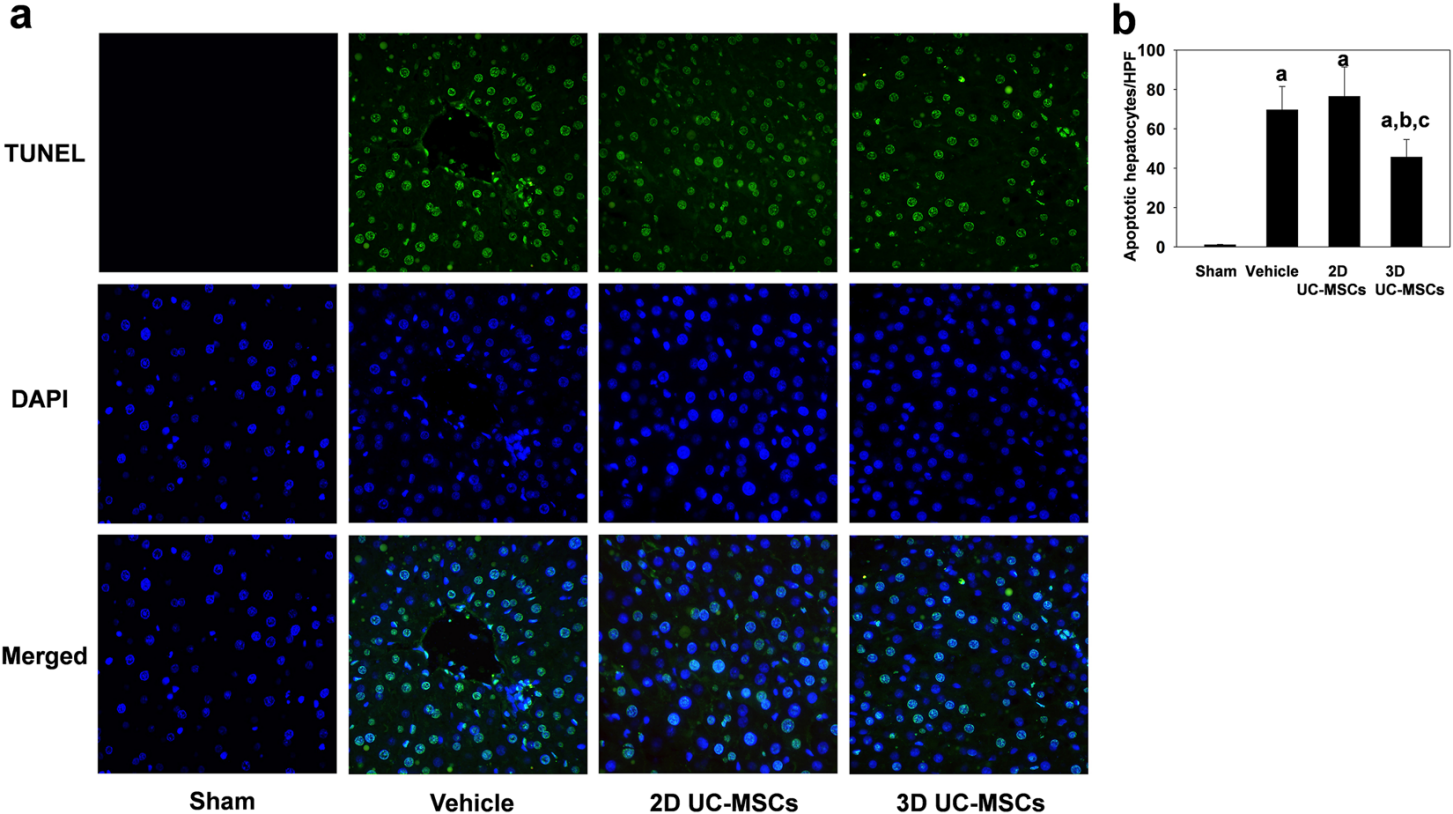
3D UC-MSCs attenuated hepatocyte apoptosis after hepatic IRI. (**a**) The apoptotic hepatocytes are shown with TUNEL staining (green) at 6 h after hepatic IRI (×400). The nuclei of hepatocytes were counterstained with DAPI (blue). Merged images of TUNEL and DAPI staining are shown at the bottom row of the panel. (**b**) Few hepatocytes were apoptotic in the Sham group. The hepatic IRI resulted in profound cell apoptosis. At 6 hours after hepatic IRI, the number of apoptotic hepatocytes in the 3D UC-MSCs group was significantly less than vehicle and 2D UC-MSCs treatment groups. The Data are means ± SE (n = 4–6); ^a^*P* < 0.05 compared Sham group; ^b^*P* < 0.05 compared with Vehicle group, ^c^*P* < 0.05 compared 2D UC-MSCs treatment group. Abbreviations: TUNEL, terminal deoxynucleotidyl transferase dUTP nick end labeling; DAPI, 4′,6-diamidino-2-phenylindole; HPF, high power fields (400×).

The terminal deoxynucleotidyl transferase (TdT)-mediated dUTP-biotin nick end labeling (TUNEL) staining was used to identify the apoptotic hepatic cells. As expected, few hepatocytes were apoptotic in livers of sham surgery group (Fig. 6), while many hepatocytes were undergoing apoptosis in the vehicle and UC-MSCs treatment groups at 6 hours after IRI. The number of apoptotic hepatocytes was 45.60 ± 9.04 hepatocytes/high power fields in the 3D UC-MSCs treatment group, which was significantly less compared with 69.60 ± 11.91 hepatocytes/high power fields in the vehicle treatment group and 76.40 ± 14.99 hepatocytes/high power fields in the 2D UC-MSCs treatment group (*P* < 0.05).

### 3D UC-MSCS treatment inhibited tumor necrosis factor-α (tnf-α) mrna expression and increased il-6 mrna expression in iri liver tissues

**Figure 7:**
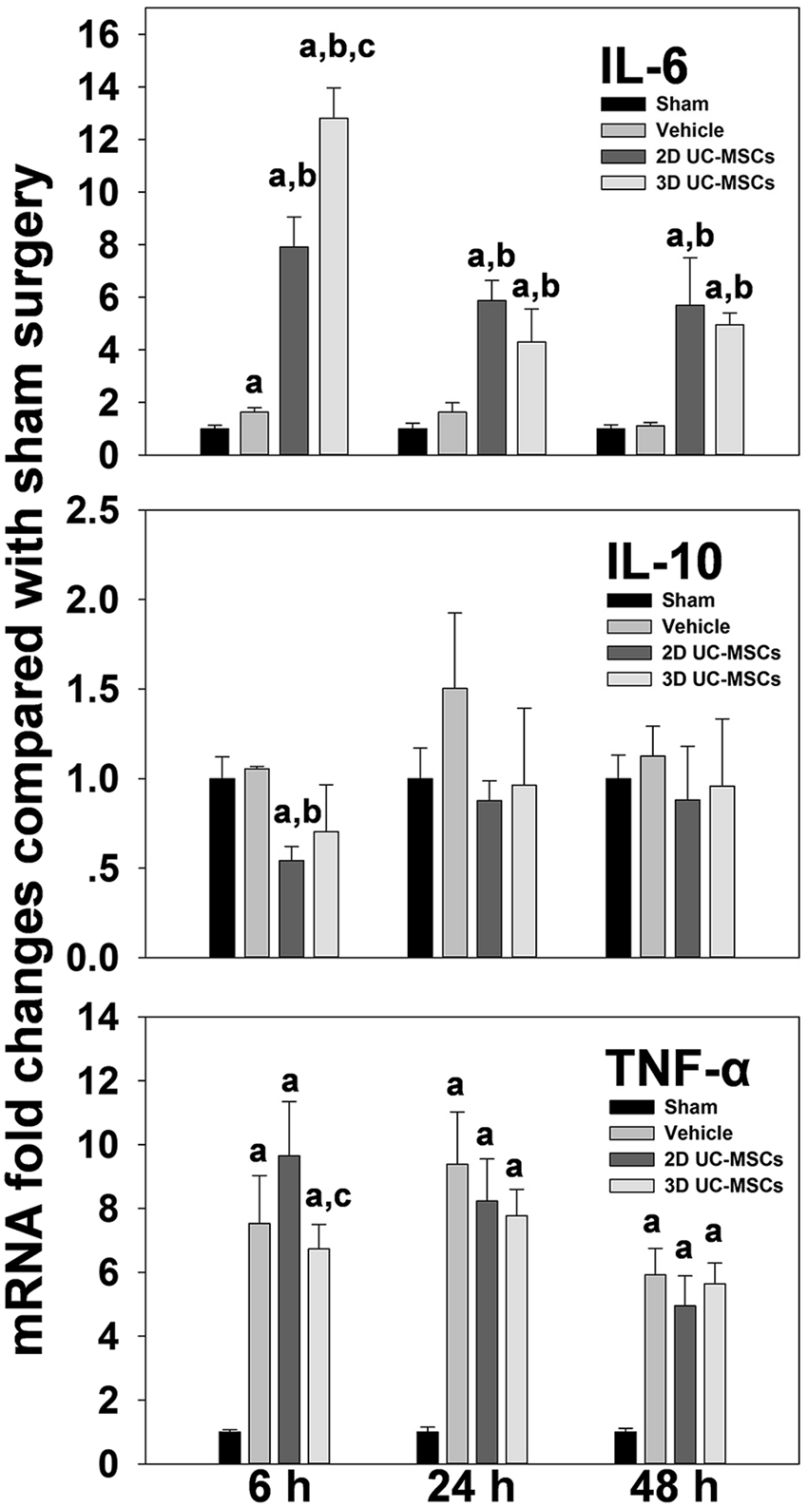
Effect of UC-MSCs treatment on the expression of inflammatory genes after hepatic IRI. Animal treatments and methods are as described in materials and methods. IL-6, IL-10, and TNF-α gene expression at different time points after hepatic IRI, as determined by real-time PCR are shown. Data are means ± SE (*n* = 4–6); ^a^*P* < 0.05 compared Sham group; ^b^*P* < 0.05 compared with Vehicle group, ^c^*P* < 0.05 compared 2D UC-MSCs treatment group.

The expression of key pro-inflammatory and anti-inflammatory cytokines (TNF-α, IL-6, and IL-10) in the liver tissue after hepatic IRI was determined by real-time PCR (Fig. 7). Hepatic IRI significantly increased the expression of the TNF-α gene in all groups, but with different temporal patterns. 2D and 3D UC-MSCs treatments did not cause a significant difference in hepatic expression of TNF-α gene compared with the vehicle group, but the 3D UC-MSCs group had a lower hepatic expression of TNF-α gene compared with the 2D UC-MSCs group (6.73 ± 0.77 VS 9.65 ± 1.7, respectively; *P* < 0.05) at 6 h time point. Hepatic IRI caused a slight elevation of the IL-6 gene (1.63 ± 0.17) at 6 h time point in the vehicle treatment group, while the 2D and 3D UC-MSCs caused a dramatic increase in IL-6 gene expression at all time points after hepatic IRI compared with the vehicle group. The expression of IL-6 was significantly higher in the 3D UC-MSCs group compared with the 2D UC-MSCs group (12.81 ± 1.15 VS 7.92 ± 1.14, respectively; *P* < 0.05) at 6 h after IRI. The IL-10 gene expression did not change significantly in the 3D UC-MSCs treatment group at all time points after hepatic IRI compared with the vehicle treatment group. However, the 2D UC-MSCs group had significantly less IL-10 gene expression (0.54 ± 0.08) at 6 h time points compared with the vehicle treatment group (1.05 ± 0.03, *P* < 0.05).

## Discussion

Compared with 2D cell culture, 3D cell culture was regarded as more physiological and better preserved the *in vivo* environment and characteristics of MSCs^36^. In our study, the RNA sequencing analysis revealed that a lot of genes which participate the angiogenesis, inflammation, and inhibiting cell proliferation, were differentially regulated in 3D spheroid-cultured UC-MSCs compared with 2D-cultured UC-MSCs. This result was consistent with the previous studies of human bone marrow- and adipose tissue-derived MSCs, which showed that spheroid-culture caused a significant upregulation of angiogenetic genes, and enhanced expression of pro-inflammatory and anti-inflammatory genes by the gene microarray analysis^16,28,37,38^. Yeh *et al*., also performed a microarray analysis of human UC-MSC spheroids which were formed on chitosan membranes. In their study, the pro-inflammatory genes including IL1A, IL1B, IL33, and TNFSF13B, were upregulated, while some anti-inflammatory genes including TSG-6, and prostaglandin-endoperoxide synthase 2, also had increased expression^38^. Our RNA sequencing data, together with previous microarray studies, all proved that spheroid-culture of MSCs indeed induced an upregulation of both pro-inflammatory and anti-inflammatory genes at the transcription level. Furthermore, a very interesting finding in our study is that both the mRNA and protein of ZC3H12A, was significantly upregulated in 3D UC-MSCs compared with 2D UC-MSCs. ZC3H12A encodes a RNase, which destabilizes mRNAs encoding pro-inflammatory cytokines including IL2, IL6, CXCL1, CXCL2, and CXCL3, via its 3′ untranslated regions^39,40^. This finding may suggest an enhanced anti-inflammatory modulation of 3D UC-MSCs at the post-transcription level, which may be a new mechanism that leads 3D UC-MSCs to exhibit a more potent anti-inflammatory effect in *in vitro* and *in vivo* studies.

The MSCs could be either pro-inflammatory or anti-inflammatory in immune modulation. Recent studies have shown that in the presence of LPS or low levels of TNF-α and interferon-γ, MSCs may adopt a pro-inflammatory phenotype and secrete chemokines such as MIP-1, MIF-1, and CXCLs, which recruit neutrophils, lymphocytes, and macrophages to enhance inflammation^25,26,41^. As the chemotaxis plays a critical role in MSC-mediated inflammatory response, the differences in chemokines production between 2D and 3D UC-MSCs were investigated in this study. The results showed that 2D UC-MSCs secreted a lot of chemokines, among which MIF, MCP-1, and SCGF-β were of the highest concentration. 3D culture dramatically reduced the production of these chemokines. These findings indicate that 3D culture may turn UC-MSCs into an anti-inflammatory phenotype by reducing chemokine production, and therefore, may be more suitable for treating inflammation associated diseases. UC-MSCs also produce trophic factors. In this study, the 2D UC-MSCs secreted large amount of HGF, SCGF-β, G-CSF, and M-CSF, but VEGF was almost undetectable. The 3D UC-MSCs secreted a high level of VEGF but other growth factors were significantly reduced compared with 2D UC-MSCs. These results are consistent with our RNA-sequencing results which showed that the 3D culture not only induced an upregulation of genes associated with hypoxia and angiogenesis, but also caused a significantly inhibition of cell proliferation. The increased VEGF production in 3D UC-MSCs may benefit their treatment for ischemic damages.

In the animal hepatic IRI model, we administrated 2D and 3D UC-MSCs by intraperitoneal injection, which prevented MSCs from entrapping in lungs through intravenous infusion in previous studies^22^. The *in vivo* imaging and immunostaining revealed that both 2D and 3D UC-MSCs could migrate to the liver after administration. The pathological studies showed that 2D UC-MSCs could not prevent hepatic IRI. The plasma transaminase levels of 2D UC-MSCs group were even slightly higher than vehicle group at 6 h time point (*P* > 0.05). One reason for the failure of 2D UC-MSCs treatment might be that the duration of hepatic ischemic injury was 90 minutes in our model, whereas the models described in many other studies used 30 to max 60 minutes of ischemia^18–21,24,42^. The damage in our study could be too severe to detect the effects of 2D UC-MSCs. Another reason might be that the 2D UC-MSCs secreted much more chemokines compared with 3D UC-MSCs, which could even aggravate the IRI induced hepatic inflammation. A similar finding was reported by Fouraschen, *et al*.^43^. The mesenchymal stem cell-derived factors did not attenuate liver injury in a mouse model of 90 min hepatic IRI, but effectively prevented liver injury after 60 minutes of hepatic ischemia with 50% partial hepatectomy^43^.

The plasma transaminase levels and the pathological studies showed that 3D UC-MSCs could significantly attenuate the IRI injury by reducing the hepatic inflammation and apoptosis compared with vehicle and 2D UC-MSCs. These beneficial results could be explained by the facts that 3D UC-MSCs reduced their secretion of chemokines, enhanced the production of anti-inflammatory properties, and promoted the secretion of angiogenetic factors, such as VEGF. The proliferation of hepatocytes at the none-necrotic areas of the liver was not increased in the 3D treatment group compared with the 2D and vehicle groups according to the PCNA staining. This may be due to that 3D UC-MSCs do not secret other trophic growth factors, such as HGF, as much as 2D UC-MSCs do, or less compensatory regeneration response due to less hepatic damage compared with the vehicle and 2D MSCs treated animals. The hepatic expression of several inflammation-associated genes was investigated in this study. The results showed that hepatic IL-6 mRNA expression was significantly up-regulated in both 2D and 3D UC-MSCs treatment groups at all time points compared with the vehicle group. IL-6 is a multifunctional cytokine with well-defined pro- and anti-inflammatory properties. IL-6 induces anti-inflammatory and regenerative signaling pathways after binding to its membrane-bound receptor, which is only expressed on hepatocytes and certain subpopulations of leukocytes. The pro-inflammatory roles of IL-6 have been attributed to the trans-signaling pathway when IL-6 binds to soluble forms of the IL-6 receptor and interacted with the other cells^44^. Previous studies showed that IL-6 reduced the hepatic IRI both in normal and obese rodents^45,46^. Although a similar trend of hepatic IL-6 mRNA expression was found in 2D and 3D UC-MSCs treatment groups, the 2D UC-MSCs treatment group had significantly higher TNF-α mRNA expression compared with the 3D UC-MSCs group and significantly lower hepatic IL-10 mRNA expression compared with the vehicle group at 6 h time point. These findings indicate that 2D UC-MSCs had multiple effects on hepatic IRI, including the negative effects such as promoting inflammation and impairing their therapeutic effects.

Taken together, the 3D culture induced profound changes in gene transcription of UC-MSCs. The genes participating angiogenesis were upregulated, while genes promoting proliferative were downregulated in 3D UC-MSCs compared with 2D UC-MSCs. Although both pro-inflammatory and anti-inflammatory genes were upregulated, 3D UC-MSCs significantly reduced their chemokines production in secretome. This could be partially explained by the upregulation an RNases (ZC3H12A), which control the pro-inflammatory cytokine transcript turnover. The 3D UC-MSCs produced more angiogenetic trophic cytokines in the secretome compared with the 2D culture. 3D UC-MSCs could attenuate hepatic IRI in rats by inhibiting hepatic inflammation and apoptosis. Our data show that 3D culture may be a useful strategy for UC-MSCs treatment of hepatic IRI.

## Methods

### Cell culture

UC-MSCs were obtained from the National Engineering and Research Center of Human Stem Cell, and their isolation and culture were described in the previous study^47^. Briefly, UC-MSCs were recovered from liquid nitrogen and cultivated in cell culture dishes with MSCs culture system: DMEM-high glucose (Gibco-BRL, USA) with 10% fetal bovine serum (Gibco-BRL, USA) and 10ng/ml bFGF (Gibco-BRL, USA). The cell culture incubator was set to 37 °C and 5% CO2. The UC-MSCs at passage 3 were used for subsequent experiments. For 2D culture, 2.5 × 10^5^ MSCs were planted in the six-well plates in 2 ml medium. To form the sphere, MSCs were digested with tryptase (Gibco-BRL, USA) and collected. The collected MSCs were resuspended in the culture system with the concentration of 6250 cells/μl. A drop of 40 μl was placed on the inverted lid of a cell culture dish. The lid was then rapidly reinverted onto the culture dish that contained PBS to prevent evaporation of the drop. The cells were collected for transplantation and RNA sequencing after 72 hours. To analyze the secreted cytokines from MSCs, the medium was changed to serum-free DMEM at 48 hours, and the supernatant was collected at 72 hours. Altogether 3 dishes of cells and 3 conditioned media from 2D- and 3D-cultured UC-MSCs, respectively, were collected for RNA sequencing and cytokine immunoassay.

### Animals

Male Sprague Dawley rats weighing 250–300 g were purchased from the department of animal research, Xiangya Medical College, Central South University, China. Rats were housed in a pathogen-free facility with a 12-hour light-dark cycle. Food and tap water were allowed ad libitum. Animal surgery protocols were approved by the Animal Care and Use Committee of the Central South University, and the animal experiments were performed in adherence to National Institutes of Health guidelines for the use of laboratory animals. All the surgeries were done between 9 to 12 am.

### Hepatic IRI animal model

A partial hepatic IRI model was induced as described previously with minor changes^48^. Briefly, rats were completely anesthetized with isoflurane. After opening the abdomen and dissecting the interlobular ligaments, all structures in the portal triad to the left and median liver lobes were occluded using a microvascular clamp for 90 min. Rats received intraperitoneal injections of vehicle (1 ml saline, n = 6), 2D UC-MSCs (3 × 10^6^ per rat, n = 6), or 3D UC-MSCs (3 × 10^6^ per rat, n = 6) immediately after reperfusion. Sham control rats underwent the same protocol without vascular occlusion.

### Multiplex-Microbead immunoassay

A multiplex-biometric immunoassay containing fluorescent microspheres conjugated with specific monoclonal antibodies was performed to analyze cytokine levels in the conditioned culture medium following manufacturer’s instruction (Bio-Plex Pro^TM^ Human Cytokine 21-Plex and Bio-Plex Pro^TM^ Human Cytokine 27-plex Panel, Bio-rad, USA). The cytokines selected from 21-plex for testing were as follows: HGF, IFN-α, LIF, M-CSF, MIF, SCF, SCGF-β, and SDF-1α. The cytokines selected from 27-plex for testing were as follows: bFGF, G-CSF, granulocyte-macrophage colony-stimulating factor, MCP-1, platelet-derived growth factor-BB, and VEGF. The data were processed using the Luminex data collection software (vertion6.1).

### RNA sequencing

RNA of 2D- and 3D-cultured UC-MSCs were isolated with TRIzol reagent (Thermo Scientific, USA), then quantified with a nanodrop spectrophotometer (Thermo Scientific, USA). RNA sequencing was carried out by the Shenzhen BGI Genomics Institute following standard protocols. Briefly, the total RNA samples were treated with DNase I to degrade any possible DNA contamination. Then, the mRNA was enriched by using the oligo(dT) magnetic beads. After mixing with the fragmentation buffer, the mRNAs were fragmented into short fragments. The first strand of cDNA was synthesized using random hexamer-primers. Buffer, dNTPs, RNase H and DNA polymerase I were added to synthesize the second strand. The double strand cDNAs were purified with magnetic beads. End reparation and 3′-end single nucleotide A (adenine) addition were then performed. Finally, sequencing adaptors were ligated to the fragments. The fragments were enriched by PCR amplification. Agilent 2100 Bioanalyzer and ABI StepOnePlus Real-Time PCR System were used to qualify the sample library. The library products were sequenced via Illumina HiSeqTM 2000. Standard bioinformatics analysis was performed by the BGI Genomics Institute.

### Western Blotting

Western blotting was performed as described previously^49^. In brief, cells were harvested and lysed in 1× RIPA buffer (Sigma, St. Louis, MO) and protein was quantified using the Bradford reagent (BioRad, Marnes-la-Coquette, France). Cell lysates were loaded on SDS-polyacrylamide gels and Western blotting was performed using standard protocols with antibodies against ZC3H12A (GeneTex, CA, USA), and β-ACTIN (Sigma). After reaction with secondary antibodies, the antibody-bound proteins were detected using an ECL Western blotting kit (GE Healthcare Life Sciences, PA).

### *In vivo* imaging of MSCs

MSCs were labeled with 3.5 μg/mL of 1,1′-dioctadecyltetramethyl indotricarbocyanine Iodide (DiR, PerkinElmer, USA) by addition of the dye into cells suspended in PBS. After 30 min incubation at 37 °C, cells were extensively washed with PBS twice and injected into the rat abdomen after reperfusion. The animals were then imaged after surgery using the FMT 4000™ fluorescence tomography *in vivo* imaging system (PerkinElmer, USA).

### Biochemical analyses and histology

Liver sections (5 μm) were stained with hematoxylin and eosin for histological analysis. The hepatic damage was evaluated using the Suzuki score^50^. The cell cycle progression (per 1,000 hepatocytes) was estimated using specific PCNA staining patterns and cell morphology as described previously^51^. Serum alanine transaminase levels were determined using standard kits (Thermotrace, Melbourne, Australia). Apoptotic cells were determined by the TUNEL staining *in situ* cell death detection kit (Fluorescein, Roche, Mannheim, Germany). Neutrophil infiltration was assessed by staining tissue sections for chloroacetate esterase using the naphthol AS-D chloroacetate esterase kit (Sigma, St. Louis, MO).

### RNA isolation and real-time PCR

RNA extraction and real-time PCR were performed as described previously^51^. The PCR primers for TNF-α, IL-6, and IL-10 (Table 1) were designed using Primer 3 (Whitehead Institute for Biomedical Research, Cambridge, MA). Primers were designed to cross exons to ensure that only cDNA and no genomic DNA was amplified. The comparative Ct method was used to determine fold differences between samples and the calibrator gene (β-actin). The comparative Ct method was used to determine the amount of target, normalized to an endogenous reference (β-actin) and relative to a calibrator (2^−ΔΔCt^).

**Table 1:** Primer Sequences for IL-6, IL-10, TNF-α, and β-actin.

Gene	Forward primer (5′-3′)	Reverse primer (5′-3′)
IL-6	TTCGGTCCAGTTGCCTTCT	GGTGAGTGGCTGTCTGTGTG
IL-10	TGGGGGAGAACCTGAAGA	ATGGCTTTGTAGATGCCTTTC
TNF-α	CTCCTCACCCACACCATCA	GGAAGACCCCTCCCAGATAG
β-actin	GGCTCCCAGCACCATGAA	AGCCACCGATCCACACAGA

### Statistical analyses

2-tailed Student’s t-test or one-way analysis of variance (ANOVA) was used for the determination of statistical significance among treatment groups, as appropriate. Differences were considered significant at *P* < 0.05. Results were reported as means ± SE (n = 3–6). For RNA sequencing data, gene expression levels were reported as fragments per kilobase million. Differences were considered significant at probability ≥0.8 and |Log_2_ (fold of gene expression change)|≥1. The DAVID (database for annotation, visualization and integrated discovery) software was used for gene functional classification and gene ontology analysis.

## Electronic supplementary material

Supplementary table and figure
