# Supplementary material for: Spheroid-cultured human umbilical cord-derived mesenchymal stem cells attenuate hepatic ischemia-reperfusion injury in rats
Source: Sci Rep. 2018 Feb 6;8:2518. doi: 10.1038/s41598-018-20975-0 (PMC5802716; doi:10.1038/s41598-018-20975-0)
Supplement: Supplementary file 1 — Supplementary table and figure [file 41598_2018_20975_MOESM1_ESM.pdf]

Spheroid-cultured human umbilical cord-derived mesenchymal stem cells attenuate hepatic ischemia-reperfusion injury in rats

Yi Sun[1,2,3], Yang Wang[1,2,3], Liang Zhou[4], Yizhou Zou[5], Gengwen Huang[6], Ge Gao[7], Shi Ting[7], Xiong Lei[6] and Xiang Ding[8,*]

[1] Institute of Reproductive and Stem Cell Engineering, School of Basic Medical Science, Central South University, Changsha, 410078, China.

[2] National Engineering and Research Center of Human Stem Cell, Central South University, Changsha, 410250, China.

[3] Key Laboratory of Stem Cells and Reproductive Engineering, Ministry of Health, Changsha, 410250, China.

[4] Department of Ophthalmology, Second Xiangya Hospital, Central South Univerisity, Changsha, 410011, China.

[5] Department of Immunology, Xiangya School of Medicine, Central South University, Changsha, 410078, China.

[6] Department of General Surgery, Xiangya Hospital, Central South University, Changsha, 410078, China.

[7] Department of Laboratory Medicine, Xiangya School of Medicine, Central South University, Changsha, 410013, China.

[8] Department of Organ Transplantation, Xiangya Hospital, Central South University, Changsha, 410078, China.

*Corresponding Author: xiangding@csu.edu.cn

Supplementary Table 1. The differential regulated genes enriched in the biological process of positive regulation of angiogenesis

GeneID	Symbol	FPKM1 (3D UC-MSCs)	FPKM2 (2D UC-MSCs)	log2 ratio of FPKM1/FPKM2	Probability
694	BTG1	55.91666667	10.00333333	2.482797543	0.90884021
3576	IL8	3089.476667	787.1533333	1.97264588	0.899162287
133	ADM	141.3366667	9.623333333	3.876455278	0.948887386
51129	ANGPTL4	426.17	7.416666667	5.844514298	0.982916211
1116	CHI3L1	148.4766667	7.656666667	4.277375966	0.958643356
57105	CYSLTR2	4.91	1.2	2.032688619	0.818027299
1545	CYP1B1	8.043333333	1.01	2.993438217	0.886267062
3162	HMOX1	87.16333333	6.516666667	3.741515263	0.945496644
3082	HGF	225.11	91.97666667	1.291290328	0.867483567
3552	IL1A	88.58	37.42	1.243171439	0.862237022
3553	IL1B	1811.013333	485.1633333	1.900254741	0.897427893
388	RHOB	132.9766667	35.26666667	1.914795993	0.895520058
7291	TWIST1	39.15333333	7.593333333	2.366329891	0.905683612
7422	VEGFA	572.1733333	157.5366667	1.860764606	0.896621399
80149	ZC3H12A	38.53666667	13.25333333	1.539876532	0.87299027

Supplementary Table 2. The differential regulated genes enriched in the inflammatory process

GeneID	Symbol	FPKM1 (3D UC-MSCs)	FPKM2 (2D UC-MSCs)	log2 ratio of FPKM1/FPKM2	Probability
604	BCL6	38.4	10.56333333	1.862041152	0.888729903
6364	CCL20	27.22	0.666666667	5.351557662	0.969717467
6349	CCL3L1	4.336666667	0.01	8.760442746	0.923582566
414062	CCL3L3	4.193333333	0.693333333	2.596476489	0.82721092
6352	CCL5	56.4	8.573333333	2.717767021	0.915387551
6354	CCL7	51.78666667	12.89666667	2.005582472	0.897714068
2920	CXCL2	82.52666667	14.62333333	2.496588164	0.910748044
2921	CXCL3	165.32	43.23666667	1.934934061	0.89627452
3576	IL8	3089.476667	787.1533333	1.97264588	0.899162287
7852	CXCR4	9.77	0.563333333	4.116297816	0.928248088
1051	CEBPB	59.68	24.48666667	1.28525114	0.862627261
929	CD14	3.476666667	0.2	4.119632847	0.856869071
1999	ELF3	7.293333333	0.08	6.510434522	0.949251609
1969	EPHA2	11.11333333	35.21666667	-1.663966758	0.88313648
2353	FOS	14.84666667	2.69	2.46446098	0.89372496
8111	GPR68	30.08333333	6.78	2.149607253	0.896213816
5971	RELB	22.51333333	6.546666667	1.78194725	0.880578247

7130	TNFAIP6	57.43666667	13.42333333	2.097229053	0.900003469
4982	TNFRSF11B	87.02	24.76666667	1.812947307	0.89240682
7133	TNFRSF1B	8.243333333	2.166666667	1.927750616	0.850972128
27242	TNFRSF21	37.41666667	11.93666667	1.648281021	0.883153823
8771	TNFRSF6B	9.606666667	31.36333333	-1.706971064	0.883370623
10318	TNIP1	174.6166667	74.05333333	1.237554685	0.864517752
135	ADORA2A	11.10666667	0.896666667	3.630710323	0.921093709
301	ANXA1	62.17	244.6733333	-1.976566388	0.898364466
650	BMP2	8.37	0.596666667	3.810230536	0.915656382
623	BDKRB1	188.1266667	77.91333333	1.27176222	0.866078707
624	BDKRB2	71.17	22.08	1.688529064	0.8884524
1116	CHI3L1	148.4766667	7.656666667	4.277375966	0.958643356
9734	HDAC9	4.786666667	0.79	2.599096785	0.840140833
3552	IL1A	88.58	37.42	1.243171439	0.862237022
3553	IL1B	1811.013333	485.1633333	1.900254741	0.897427893
3656	IRAK2	29.76333333	4.75	2.647536691	0.910886796
11009	IL24	10.12666667	0.193333333	5.710925159	0.956562083
3569	IL6	489.4766667	35.79666667	3.773342931	0.948098236
3988	LIPA	9.603333333	22.92	-1.254999884	0.849463205
11343	MGLL	12.14333333	30.19	-1.313906262	0.858065803
4791	NFKB2	52.84666667	19.14333333	1.464970415	0.871299235

5806	PTX3	8.806666667	108.8733333	-3.627910763	0.94350209
8605	PLA2G4C	6.293333333	2.106666667	1.578862301	0.815165548
55848	PLGRKT	1.75	5.926666667	-1.759865996	0.820169277
5742	PTGS1	5.303333333	0.813333333	2.704980783	0.850824705
5743	PTGS2	1473.01	259.1333333	2.507000713	0.913991363
8767	RIPK2	82.78666667	25.67333333	1.689127809	0.889094126
8986	RPS6KA4	4.273333333	9.683333333	-1.180141902	0.809424701
8482	SEMA7A	17.73333333	52.70666667	-1.571521709	0.877083442
12	SERPINA3	9.176666667	1.966666667	2.22221775	0.868723659
6335	SCN9A	0.136666667	3.12	-4.512812715	0.854518966
6382	SDC1	4.6	27.34333333	-2.571483361	0.904790398
7057	THBS1	68.04666667	150.49	-1.145071227	0.858369322
9473	THEMIS2	8.36	2.22	1.912943266	0.851249631
80149	ZC3H12A	38.53666667	13.25333333	1.539876531	0.87299027
85463	ZC3H12C	18.09333333	5.336666667	1.761447413	0.875878037

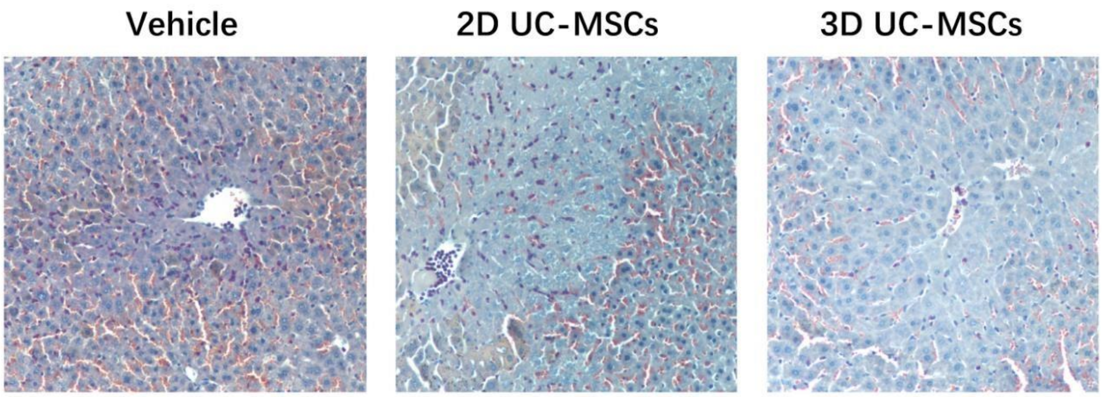

Supplementary figure 1. The representative picture (×400) of chloracetate esterase staining of neutrophils (purple) at 24 h after hepatic IRI. Significantly less neutrophils infiltrated into the liver in 3D UC-MSCs treatment compared with vehicle and 2D UC-MSCs treatment groups.

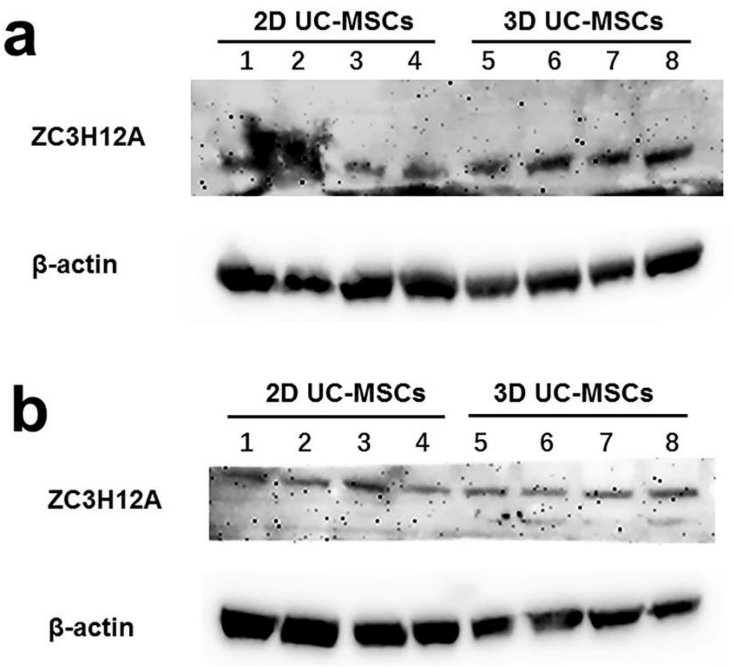

Supplementary figure 2. Two independent Western Blotting experiments were performed to confirm the expression of ZC3H12A protein in UC-MSCs. The cell culture and Blotting were described in Methods. The ZC3H12A protein levels were normalized to corresponding β-actin levels. Both experiments showed that the ZC3H12A level was significantly higher in 3D UC-MSCs group compared with 2D UC-MSCs group. a) The full-length gel of first Western Blotting experiment was shown, the No 2 sample of 2D UC-MSCs was excluded from statistics due to too much background. b) The second independent Western Blotting experiment was performed with the protein samples from another batch of recovered and cultured UC-MSCs cells. The result also showed that the ZC3H12A protein level was higher in 3D UC-MSCs when normalized to the β-actin level.
